# The involvement of TRPC3 channels in sinoatrial arrhythmias

**DOI:** 10.3389/fphys.2015.00086

**Published:** 2015-03-25

**Authors:** Yue-Kun Ju, Bon Hyang Lee, Sofie Trajanovska, Gouliang Hao, David G. Allen, Ming Lei, Mark B. Cannell

**Affiliations:** ^1^Department of Physiology, School of Medical Sciences, Bosch Institute, University of SydneySydney, NSW, Australia; ^2^Department of Pharmacology, University of OxfordOxford, UK; ^3^Department of Physiology and Pharmacology, University of BristolBristol, UK

**Keywords:** TRPC3, receptor-operated Ca^2+^ entry, store-operated Ca^2+^ entry, arrhythmias, sinoatrial node

## Abstract

Atrial fibrillation (AF) is a significant contributor to cardiovascular morbidity and mortality. The currently available treatments are limited and AF continues to be a major clinical challenge. Clinical studies have shown that AF is frequently associated with dysfunction in the sino-atrial node (SAN). The association between AF and SAN dysfunction is probably related to the communication between the SAN and the surrounding atrial cells that form the SAN-atrial pacemaker complex and/or pathological processes that affect both the SAN and atrial simultaneously. Recent evidence suggests that Ca^2+^ entry through TRPC3 (Transient Receptor Potential Canonical-3) channels may underlie several pathophysiological conditions -including cardiac arrhythmias. However, it is still not known if atrial and sinoatrial node cells are also involved. In this article we will first briefly review TRPC3 and IP_3_R signaling that relate to store/receptor-operated Ca^2+^ entry (SOCE/ROCE) mechanisms and cardiac arrhythmias. We will then present some of our recent research progress in this field. Our experiments results suggest that pacing-induced AF in angiotensin II (Ang II) treated mice are significantly reduced in mice lacking the TRPC3 gene (TRPC3^−/−^ mice) compared to wild type controls. We also show that pacemaker cells express TRPC3 and several other molecular components related to SOCE/ROCE signaling, including STIM1 and IP_3_R. Activation of G-protein coupled receptors (GPCRs) signaling that is able to modulate SOCE/ROCE and Ang II induced Ca^2+^ homeostasis changes in sinoatrial complex being linked to TRPC3. The results provide new evidence that TRPC3 may play a role in sinoatrial and atrial arrhythmias that are caused by GPCRs activation.

## Introduction

### The role of intracellular Ca^2+^ in sinoatrial arrhythmias

In the normal heart, pacemaker cells in the sinoatrial node (SAN) generate spontaneous membrane depolarizations that trigger action potentials, which then propagate through the conduction system to initiate atrial and ventricular cell depolarization and contraction. In contrast to normal pacemaker activity, abnormal arrhythmogenic electrical activity can arise in ectopic sites due to impulse re-entry or abnormal spontaneous membrane depolarizations (Nattel, 2002).

SAN and the surrounding atria form the SAN-Atrial pacemaker complex, which can provide substrates for re-entrant activity that can lead to AF (Sanders et al., 2004; Fedorov et al., 2010). Dysfunction of pacemaker ion channels, including altered HCN4 channels, are associated with familial tachycardia-bradycardia syndrome and atrial fibrillation (Duhme et al., 2013). While it is clear that cardiac arrhythmias, including SAN dysfunction and atrial fibrillation (AF), are multifactorial there is increasing experimental evidence for abnormal Ca^2+^ handling being a key factor (Dobrev and Nattel, 2008; Yeh et al., 2008) and is the focus of this paper.

**Figure 1:**
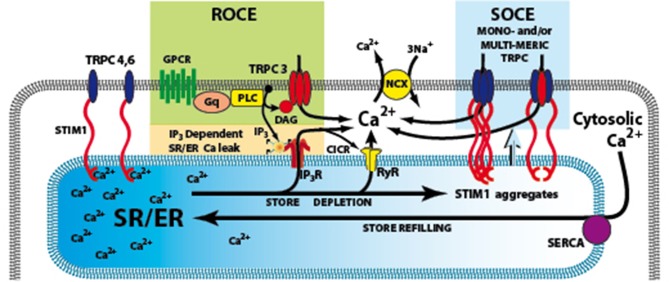
**Signaling pathways involved in the activation of TRPC3**. The G-protein coupled receptor (GPCRs) activates phospholiase C (PLC), resulting in generation of IP3 and diacylglycerol (DAG). IP3 activates its receptor that leads to Ca^2+^ release from SR/ER and the depletion of SR/ER Ca^2+^ store. The Ca^2+^ content change in the store can be sensed by STIM1, the ER Ca^2+^ sensor and cause store-operated Ca^2+^ entry (SOCE) through TRPC3 channels. An additional or alternate possibility is that DAG can directly activate TRPC3, and produce receptor-operated Ca^2+^ entry (ROCE). GPCRs, G-protein coupled receptors; IP3R, inositol 1,4,5-trisphosphate receptors; PLC, Phospholipase C; ROCE, Receptor–operated Ca^2+^ entry; SOCE, store-operated Ca^2+^ entry; SR/ER, sarco- endo-plasmic reticulum; STIM1, stromal interacting molecule 1; TRPC3,4,6 canonical transient receptor potential channel types 3,4,6; NCX Na/Ca exchange.

The most prominent Ca^2+^-dependent ionic current during pacemaker activity is the Na/Ca exchange (NCX) current which not only contributes to pacemaker current(s) but may also produce arrhythmogenic electrical activity (Sipido et al., 2006) which is related to abnormalities in Ca^2+^ handling leading to abnormal NCX currents (Hove-Madsen et al., 2004; Vest et al., 2005). Spontaneous Ca^2+^ release events or “leak” via the ryanodine receptor (RyR), the major cardiac SR Ca^2+^ release channel, will produce depolarizing NCX current and contribute spontaneous membrane depolarization(s) to feed the genesis of AF (for review see Greiser et al., 2011; Wakili et al., 2011). In addition, AF has also been linked to Ca^2+^ release via a second class of SR Ca^2+^ release channel, inositol 1,4,5-*tris*phosphate receptor family (IP_3_Rs) (Woodcock et al., 2000; Mackenzie et al., 2002; Li et al., 2005; Berridge, 2009). Activation of IP_3_Rs by IP_3_ is linked to the activation of G-protein coupled receptors (GPCRs) and the phospholipase C (PLC)-IP_3_ signaling pathway (see Figure 1). Activation of GPCRs by agonists such as angiotensin II (Ang II) or endothelin-1 (ET-1) can cause SAN dysfunction (Neef et al., 2010) and AF (Woodcock et al., 2000; Mackenzie et al., 2002; Li et al., 2005).

Previously, we reported that in mouse SAN, there is a substantial sarcolemmal Ca^2+^ influx upon SR Ca^2+^ store depletion, a phenomenon known as “store-operated Ca^2+^ entry” (SOCE) (Ju et al., 2007; Ju and Allen, 2007). We also showed that type II IP_3_R are functionally expressed and affect Ca^2+^ handling and pacemaker activity in the mouse SAN and whose activation would lead to store depletion (Ju et al., 2011, 2012). The linkage between IP_3_R activation and SOCE has proven elusive, but a potential candidate, was found in HEK 293 cells expressing the Transient Receptor Potential Canonical-3 (TRPC3) channel (see below) (Kiselyov et al., 1998).

### TRPC3, SOCE/ROCE, and STIM1

Studies on the transient receptor potential (TRP) gene from *Drosophila* showed that it encodes a PLC-activated Ca^2+^ permeable channel. Subsequently, seven homologs of TRP channels have been identified in mammals, termed TRPC1 to TRPC7 (Montell et al., 2002). TRPCs are thought to be strong candidates for the elusive SOCE pathway (for review see Salido et al., 2009). We have found that the SAN expresses all TRPC subtypes except TRPC5 (Ju et al., 2007). Among them, TRPC3 was the only subtype located in the surface membrane of pacemaker cells, making this isoform the strongest candidate for the SOCE channel in SAN (Ju et al., 2007).

Ca^2+^ influx can also be triggered as a direct consequence of PLC activation and production of diacylglycerol (DAG) that is independent of SR Ca^2+^ store depletion and forms a “receptor-operated Ca^2+^ entry” (ROCE) pathway (Mohl et al., 2011) (Figure 1). ROCE pathways may be closely related to SOCE because PLC activation can be enhanced by IP_3_-induced store depletion. TRPC channels are non-selective Ca^2+^-permeable cation channels which are activated by diacylglycerol (DAG) liberated from the plasma membrane, triggered by agonist binding to G protein—coupled receptors, such as angiotensin II and endothelin-1 receptors. Treatment with antisense RNA, to inhibit translation of endogenous TRPC3 mRNA, reduces Ca^2+^ influx activated either by receptor stimulation or passive store depletion (Wu et al., 2004; Worley et al., 2007). Therefore, it is possible that TRPC3 is involved in both SOCE and ROCE (see Figure 1) (for review see Birnbaumer, 2009).

Another important breakthrough in SOCE/ROCE research was the identification of a new component of SOCE called stromal interacting molecule 1 (STIM1) (Roos et al., 2005). STIM1 functions as an ER/SR Ca^2+^ sensor, probably via a STIM1 Ca^2+^ binding EF hand inside the SR/ER lumen. The activation of SOCE requires STIM1 migration in the SR/ER membrane where it interacts with other molecular components of the SOCE system (Lewis, 2007). In addition, conformation coupling between IP_3_R and TRPC3 has been suggested as a mechanism of activation of TRPC3 (Kiselyov et al., 1998) and a region in the C terminus of TRPC3 has been shown to interact with IP_3_ receptor as well as calmodulin (Calmodulin/IP_3_ receptor-binding region (Zhang et al., 2001).

TRPC3 in cardiac cells provides a Ca^2+^ entry pathway that is connected to pathological signaling of the heart such as in cardiac hypertrophy (Onohara et al., 2006; Eder and Molkentin, 2011). However, there is little functional data on the role of TRPC3 in pacemaker and atrial cells to date. In the present study, we present new data suggesting that pacemaker cells expresses the molecular components of SOCE/ROCE pathways, including TRPC3, STIM1, and IP_3_R2. We present preliminary experiments using TRPC3^−/−^ mice and the specific TRPC3 channel blocker Pyr10 to show that TRPC3 appears to be able to contribute to sinoatrial and atrial arrhythmias induced by activation of GPCR Ca^2+^ signaling.

## Material and methods

### Animals

Colonies of TRPC3^−/−^ mice (Hartmann et al., 2008) and their wild-type litter mates were gifts from Prof. Housley's laboratory at University of New South Wales. The mice were deeply anesthetised with intra peritoneal pentobarbitone (1 ml/2 kg) before any procedures were carried out. All procedures on mice were performed according to the guidelines of the National Health and Medical Research Council of Australia and approved by the Institutional Ethics Committee.

### Electrophysiological studies in Langendorff-perfused hearts

Hearts were cannulated and perfused with a modified Tyrode's solution containing (in mM/L) 130 NaCl, 1.8 CaCl_2_, 1.2 MgCl, 5.4 KCl, 1.2 NaH_2_PO_4_, 12 NaHCO_3_, 11 glucose, 10 HEPES, with pH adjusted to 7.4 with NaOH. Perfusion was set at a constant flow rate of 2–2.5 ml/min and solutions were oxygenated with 95% O_2_–5% CO_2_. Electrical activity was recorded with two miniature monopolar ECG electrodes (Harvard Apparatus) placed on the atria and ventricle (Zhang et al., 2011). To determine the electrical conduction velocity of SAN and surrounding atrial tissue, custom-made electrode arrays were used to record the electrical activity from the isolated Langendorff-perfused hearts as described previously (Davies et al., 2014). To assess atrial arrhythmogenesis, Langendorff-perfused hearts from WT and KO mice were subjected to programmed electrical stimulation with three different pacing protocols as described elsewhere (Head et al., 2005; Zhang et al., 2011). Atrial tachycardia (AT) was defined as a sequence of three or more atrial depolarizations at the rate of 10 Hz or more than 10Hz. AF were characterized by irregular fibrillating waveforms. Sinus bradycardia was defined as a 30% reduction of baseline heart rate (HR) from the control condition. An arrhythmia index was calculated by the number of mice that have pacing induced arrhythmias events/the total number of mice in each group.

### The preparation of intact SAN and single pacemaker cells

Intact SANs were micro-dissected from right atria as described previously (Ju et al., 2007). The central SAN was identified from anatomical land marks, including the superior vena cava, the crista terminalis, and the interatrial septum (Marionneau et al., 2005). For physiological experiments, the intact SANs were continuously superfused with Tyrode's solution at 33°C.

### Measurement of intracellular Ca^2+^, SOCE and ROCE

The isolated SANs were loaded with membrane-permeant fluorescent Ca^2+^ indicators, indo-1-AM or Fluo-4-AM (10 μM/L), using established methods (Ju and Allen, 1998; Ju et al., 2003). To measure Ca^2+^ influx through SOCE in sinoatrial tissue, preparations were exposed to a nominally Ca^2+^ free solution with the SR CaATPase pump inhibitor, cyclopiazonic acid (CPA, 10 μM) for 15 min to deplete the SR Ca^2+^ store. SOCE then occurs upon reintroduction of Ca^2+^ to the perfusate (Ju et al., 2007). ROCE was determined by measuring the agonist-mediated increases in [Ca^2+^]_i_ (Ang II, 1 μM/L and/or 1-oleoyl-2-acetyl-sn-glycerol -OAG, 100 μM/L) (Ikeda et al., 2013).

### Immunohistochemistry

A rabbit polyclonal antibody was used to label type II IP_3_Rs (1:200; Affinity Bioreagents) and TRPC3 (1:100, Alomone Lab) in single isolated pacemaker cells. A mouse antibody to STIM1 was also used (1:100, BD Biosciences). Anti-mouse or anti-rabbit secondary antibodies (Alexa-488 anti-mouse and Alexa-594 anti-rabbit (Molecular Probes) were used as secondary antibodies. Prolong gold antifade reagent with DAPI (Molecular Probes) was used as the mounting media and to provide nuclear staining.

### Osmotic mini-pump implantation

Alzet osmotic mini-pumps were implanted subcutaneously in WT, or TRPC3^−/−^ mice. Ang II was delivered at the rate of 2 μg/g/day for 10–14 days.

### Statistics

Data are expressed as mean ± SEM, with the number of preparations as *n*. Statistical tests were either Student's paired or unpaired *t*-tests, and *P* < 0.05 was used as the limit of statistical confidence.

## Results

### Evidence for TRPC3 involvment in atrial arrhythmias

**Figure 2:**
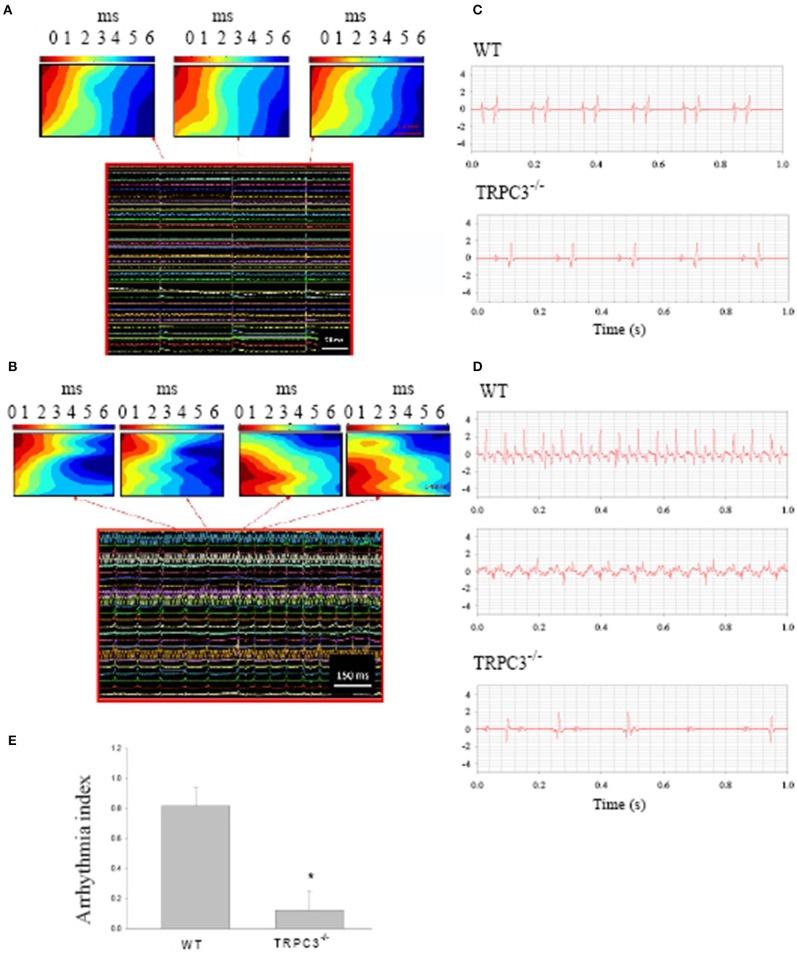
**Atrial arrhythmias induced by Ang II and pacing**. Reconstructed excitation maps of data from multi-electrode array recordings (images below) from a Langendorff-perfused isolated heart. **(A)** sinus rhythm and **(B)** pacing induced atrial tachycardia. Note in AT, the ectopic beat starts from a different site to sinus rhythms. **(C)** Representative ECG recordings from isolated heart from WT and TRPC3 ^−/−^ mice under control conditions. **(D)** Atrial tachycardia and atrial fibrillation induced by burst pacing in WT mice and atrial-ventricular conduction block recorded from a TRPC^−/−^ mouse heart respectively. **(E)** On average, the arrhythmia index was significant reduced in TRPC3KO mice (*n* = 8) compared to WT mice (*n* = 11, *P* = 0.004).

Ang II signaling pathways lead to a hypertrophy which seems to be related to TRPC3 expression (Onohara et al., 2006). To examine whether TRPC3 is involved in AF induced by Ang II, we induced AF in mice by pacing Langendorff-perfused hearts from WT and TRPC3^−/−^ KO mice which had been treated with Ang II (See Methods) over of 10–14 days. Figure 2A shows the conduction of the AP across the SAN to the atria reconstructed from the timing of the recorded electrical signals (panel below) using a 36 mini electrode array placed on the right atrium. Atrial arrhythmic events are illustrated in Figure 2B, and were induced by using a pacing protocol that varied pacing voltage and frequency. To investigate the possible role of TRPC3 in these arrhythmias, we made ECG recordings from Langendorff-perfused hearts under control condition in WT and TRPC3^−/−^ respectively, as shown in the selected recordings illustrated in Figure 2C. Electrical pacing induced AF, AT, and conduction block were recorded and analyzed in WT and TRPC3^−/−^, respectively (exemplar data shown in Figure 2D). We found that atrial arrhythmias induced by Ang II and pacing were significantly reduced in the TRPC3^−/−^ mice compared to the controls (*P* = 0.004, *n* = 11) (Figure 2E).

We previously reported that that pacemaker cells express TRPC3 and it is preferentially localized to the surface membrane (Ju et al., 2007). Therefore, the increased resistance to arrhythmias in TRPC3^−/−^ mice supports the idea of a possible involvement of TRPC3 in arrhythmogenesis. As described above, Ca^2+^ entry through TRPC3 channel activated via a SOCE/ROCE signaling pathway and STIM1 could contribute arrhythmogenic current. To investigate this possibility, we examined expression and molecular localisation of STIM1, and the possibility of molecular interaction between TRPC3, STIM1, and IP_3_R2 in isolated cardiac pacemaker cells.

### Mouse pacemaker cells express STIM1, a molecular component of SOCE /ROCE

**Figure 3:**
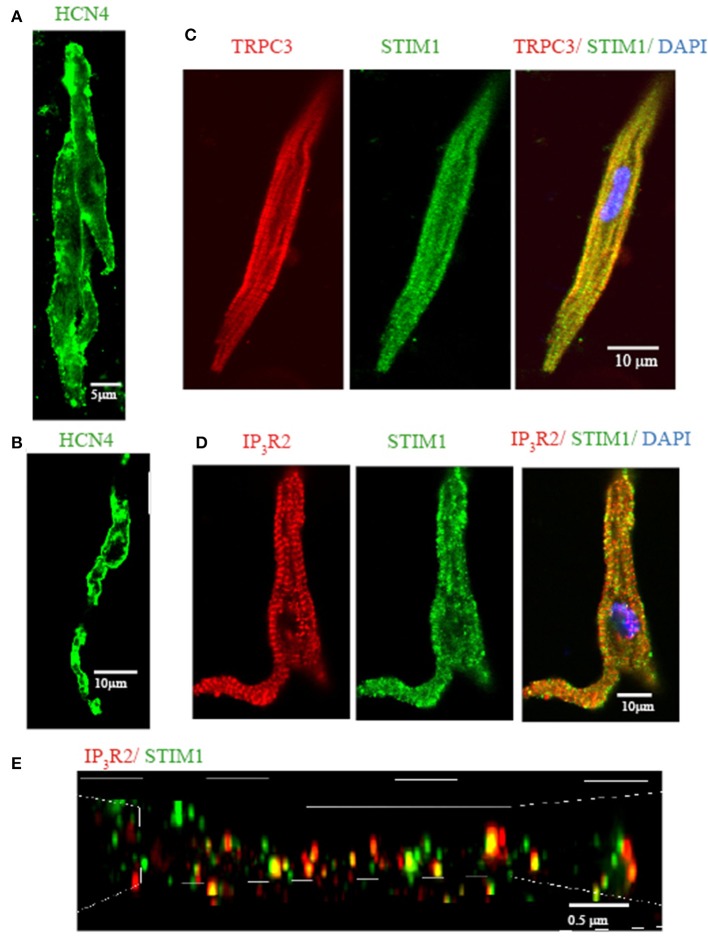
**Isolated single SAN cells express TRPC3, STIM1, and IP_3_R2**. Confocal immunofluorenscence images of isolated single pacemaker cells. Isolated group **(A)** and single **(B)** pacemaker cells were positively stained with anti HCN4 (in green). **(C)** Anti-TRPC3 in red, anti-STIM1 in green. **(D)** Anti-IP3R2 in red, anti-STIM1 in green. **(E)** 3D reconstruction image using N-SIM microscopy. Anti-IP_3_R2 in red, anti-STIM1 in green. Areas of co-localisation appear yellow due to color mixing.

Previously, we demonstrated that mouse SAN tissue expressing HCN4 mRNA also expressed TRPC3 (Ju et al., 2007). We also found that pacemaker tissue express STIM1 mRNA and protein (Liu et al., 2015). In the present study we wanted to extend these findings to the localisation of TRPC3 and STIM1 in single isolated pacemaker cells. Figure 3 shows that isolated group (Figure 3A) and single pacemaker cells (Figure 1B) were positively labeled with a HCN4 antibody (which is a common selective molecular marker for pacemaker cells). The cells isolated from same SAN region were then double labeled with TRPC3 and STIM1 antibodies, and exemplar data is shown in Figure 3C. TRPC3 (red) showed both a sarcomeric pattern as well as membrane staining while STIM1 (green) displayed both cytosolic and near membrane labeling. A previous study examined the interaction of all mammalian TRPC channels with topically expressed STIM1 and found that that the STIM1 ERM domain binds to TRPC1, TRPC4, and TRPC5 but not TRPC3, TRPC6, and TRPC7 (Worley et al., 2007). However, knock down of STIM1 significantly reduced the current carried by TRPC3 (Yuan et al., 2007). The expression of both TRPC3 and STIM1 in cardiac pacemaker cells suggested to us that TRPC3 could be involved in Ca^2+^ influx through SOCE.

We have previously reported that pacemaker cells express Type II IP_3_R (IP_3_R2) which can modulate pacemaker activity. To examine if IP_3_R and STIM1 form a signaling complex in SAN we double labeled isolated pacemaker cells with anti-IP3R2 and anti-STIM1 antibodies. Figure 3D shows that STIM1 labeling occurred both in cell periphery as well as inside the cell. IR_3_R2 was similarly distributed and there was some co-localization between these labels (yellow dots at the cell periphery in merged image). To achieve higher resolution (~100 nm), a N-SIM/N-STORM super resolution microscope was used. Figure 3E shows the higher resolution N-SIM microscope image, which revealed tighter areas of co-localization between STIM1 and IP_3_R2. Given that the necessary molecular components of SOCE/ROCE (including TRPC3, and STIM1) as well as IP_3_R are present in pacemaker cells, we next examined if SOCE can be modulated by IP_3_R activation.

### Evidence for interaction between IP_3_Rs and SOCE in mouse pacemaker tissue

To clarify if TRPC3 is involved in AF associated with GPCR activation (Figure 2), we examined whether SOCE /ROCE could be modified by GPCR activation. While we previously reported that enhanced IP_3_R Ca^2+^ signaling modulated pacemaker firing rate, it remains unknown whether IP_3_R agonists and antagonists (which can modulate heart rate) also modulate SOCE activity in SAN. Previous work on cardiac myocytes showed that overexpression of TRPC3 enhanced Ca^2+^ entry through SOCE while knock down of TRPCs inhibited SOCE (Wu et al., 2010). We therefore used the intact mouse SAN preparation loaded with the ratiometric Ca^2+^ indicator indo-1 to study the interaction between IP_3_Rs and SOCE.

**Figure 4:**
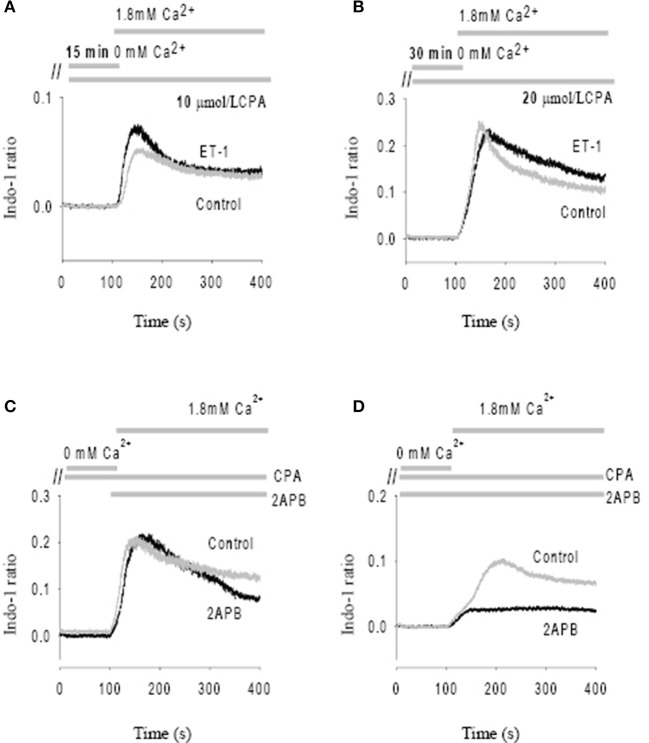
**Modulation of store-operated Ca entry (SOCE) by ET-1 and 2-APB**. SOCE was induced by using cyclopiazonic acid (CPA) and low external Ca^2+^. The addition timing of extracellular Ca^2+^ change is indicated in the top panels. **(A)** The effect of ET-1 on SOCE induced by 10 μM CPA with 15 min incubation in Ca^2+^ free Tyrode's solution. **(B)** The effect of ET-1 on SOCE induced by 20 μM CPA with 30 min incubation in Ca^2+^ free Tyrode's solution. **(C)** The effect of 40 μM 2-APB on SOCE. 2-APB was added in the same time of reintroduction of extracellular Ca^2+^ after perfusion of Ca^2+^ free Tyrode's Solution containing 10 μM CPA. **(D)** The effect of 40 μM 2-APB on SOCE. 2-APB was added into the Ca^2+^ free Tyrode's solution with 10 μM CPA during the 15 min incubation period. The gray traces represent controls; the black traces represent the treatments with ET-1 **(A,B)** or 2-APB **(C,D)**, respectively.

After initial incubation of the SAN in Ca^2+^ free Tyrode's with the SR uptake blocked with CPA (10 μmol/L), reintroduction of Ca^2+^ (1.8 mmol/L) caused a significant Ca^2+^ influx (i.e., SOCE) as shown by an increase in Indo-1 ratio (Figure 4A). Importantly, peak SOCE was increased by 63 ± 10% (*n* = 4, *P* < 0.05) in the presence of ET-1, a representative trace of which is shown in Figure 4A. Addition of the membrane permeant IP_3_ analog, IP_3_-BM (10 μmol/L) was also able to increase SOCE by 31 ± 10% (*n* = 3, data not shown). To test whether ET-1 or IP_3_-BM acted by opening IP_3_R release channels and thereby causing a greater store depletion (and hence SOCE), we examined the effect of enhancing store depletion by increasing both the CPA concentration and the incubation time in Ca^2+^ free solution. When the CPA concentration was increased from 10 μmol/L to 20 μmol/L and the incubation time in Ca^2+^ free buffer increased from 15 min to 30 min ET-1 was no longer able to further increase SOCE (Figure 4B). Similar results were found in three other SAN preparations. These results suggest that ET-1 exerts its effects on SOCE via activation of IP_3_Rs and caused Ca^2+^ release and store depletion rather than by direct stimulation of SOC channels.

Previous studies found that membrane permeant 2-APB has a direct effect on SOCE in some cell types, independent of its action as an IP_3_R antagonist. (Bootman et al., 2002) We tested this possibility by adding 40 μmol/L 2-APB at the same time that Ca^2+^ was reintroduced to the solution (Figure 4C). Ca^2+^ influx through SOCE was not inhibited by 2-APB under these conditions, suggesting that 2-APB is not working as a direct SOCE channel blockers such as gadolinium or SKF-96365 (Ju et al., 2007). However, if 2-APB was added 15 min before reintroduction of Ca^2+^, Ca^2+^ entry via SOCE was then significantly inhibited (Figure 4D). On average, Ca^2+^ entry through the SOCE channel with 2-APB decreased by 78 ± 8% (*n* = 4, *P* < 0.01). Thus, 2-APB does not serve as a classic SOCE channel blocker in pacemaker cells, but can inhibit SOCE by a slower mechanism which may involve IP_3_R2 regulation as suggested previously (Ma et al., 2000). Although the specificity of 2-APB is questionable, we found that the effect of 2-APB was diminished in IP_3_R2 KO, also supporting the involvement of IP_3_R2 in the slower onset effects of 2-APB on SOCE. These data are consistent with a previous study that suggested 2-APB blocks activation of TRPC3 indirectly via disruption of the coupling process between SOCE, TRPC and IP_3_R (Ma et al., 2002).

## Evidence TRPC3 is involved in ROCE in intact mouse sinoatrial node

TRPC3 has been implicated in not only SOCE but also ROCE (see Introduction) (for review see Birnbaumer, 2009). To further examine if Ca^2+^ influx through ROCE contributes to Ang II-induced arrhythmias we measured action potentials and Ca^2+^ influx in isolated SAN-atrial preparations treated with Ang II (1 μM) and/or OAG (100 μM), the agonists known to activate ROCE (Ikeda et al., 2013). Pyr3, a pyrazole derivative, has been suggested to be a blocker of TRPC3 (Kiyonaka et al., 2009) but it inhibits Orai1 mediated SOCE with similar potency (Salmon and Ahluwalia, 2010). Recently, a new selective blocker of TRPC3-ROCE blocker, Pyr10 has been developed and it displayed substantial selectivity for TRPC3-ROCE mediated responses over Orai1 mediated SOCE (Schleifer et al., 2012). We therefore studied the effect of Pyr10 on pacemaking and intracellular Ca^2+^ after application of Ang II and/or the DAG derivative, OAG.

**Figure 5:**
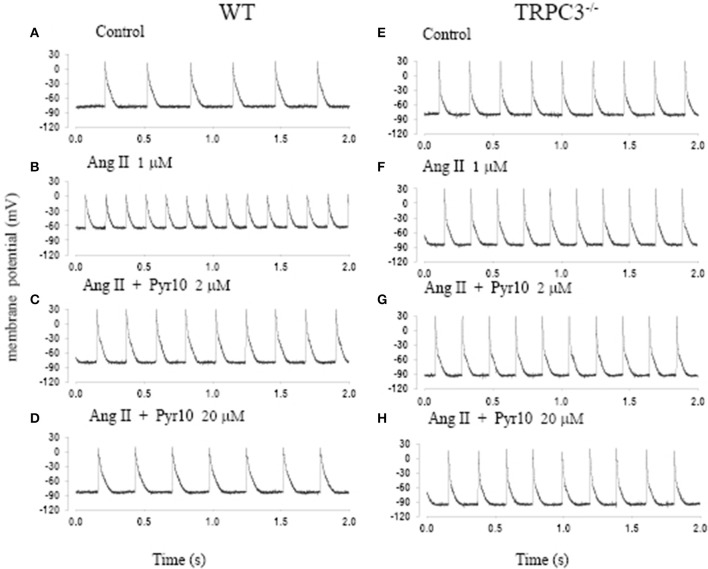
**The effect of angiotension II (Ang II) and a selective TRPC3 channel blocker Pyr10 on pacemaker action potential after application of Ang II. (A–D)** Intracellular recordings from WT mice; **(E–H**) Intracellular recordings from TRPC3^−/−^ mice with conditions as indicated in each panel.

**Figure 6:**
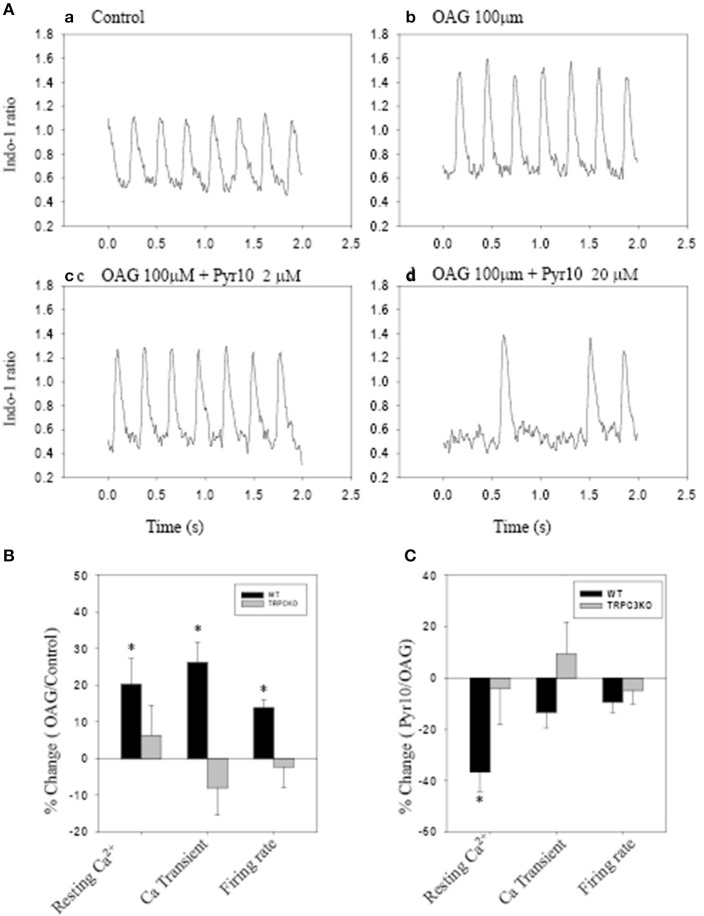
**The effect of Pyr10 on intracellular Ca^2+^ after application of 1-oleoy1-2-acyl-sn-glycerol (OAG) 100 μM**. The intact SANs were loaded with Ca^2+^ indicator indo-1. **(A,B)** show intracellular Ca^2+^ recordings from a WT mouse. **(B,C)** The statistics pool data shows percentage changes in resting Ca^2+^, Ca^2+^ transient and firing rate in WT and TRPC3 KO mice respectively. **(B)** OAG treatment against control. **(C)** 2 μM Pyr10 treatment against OAG treatment. ^*^P < 0.05.

Figure 5 shows action potentials recorded from an intact sinoatrial node preparation using conventional intracellular recording techniques. After application of 1 μM Ang II for 30 min, there was an increase in pacemaker firing rate associated with a depolarised membrane potential (Figure 5B). Application of 2 μM Pyr10 for 20 min, slowed pacemaker firing rate with maximum diastolic potential returning to the control level (Figure 5C). Application of 20 μM Pyr10 further reduced firing rate within 7 min (Figure 5D). These results suggest that the membrane depolarization caused by Ang II could be reversed by the TRPC3 channel blocker. The results also support the idea that TRPC3 could be a channel that produces an inward current after GPCR activation by Ang II (Onohara et al., 2006). Importantly, and in contrast to WT mice, the pacemaker firing rate and action potential depolarization were not affected by Ang II and this response not significantly altered by application of Pyr10 to SAN from TRPC3 KO mice (Figures 5E–H). This is consistent with pacing induced AF in mice treated with Ang II being reduced in TRPC3^−/−^ mice. (Figure 2) Therefore, these results strongly suggest that Pyr10 not only specifically blocked TRPC3 channels but that TRPC3 also contributes to pacemaker activity. To further investigate if the changes in pacemaker activity were related to Ca^2+^ entry through ROCE we examined intracellular Ca^2+^ changes caused by OAG and the effect of Pyr10. Figure 6Aa shows a representative intracellular Ca^2+^ signal recorded from a WT intact sinoatrial preparation loaded with the Ca^2+^ indicator indo-1. Both resting Ca^2+^ and Ca^2+^ transients were increased after 20 min application of 100 μM OAG (Figure 6Ab). Resting Ca^2+^ returned to the control level associated with a slowed firing rate after application of 2 μM Pyr10 for 15 min (Figure 6Ac). Further slowing and irregular pacemaker activity was apparent when the concentration of Pyr10 was increased to 20 μM for 15 min as shown in Figure 6Ad. On average, resting Ca^2+^ increased by 20.2 ± 6.9% (*P* = 0.023, *n* = 5); the Ca^2+^ transient also increased by 26.1 ± 5.6% (*p* = 0.003, *n* = 5) and was associated with a 13.9% increase in pacemaker firing rate (*p* = 0.005, *n* = 5,) in response to OAG treatment. There no significant changes by OAG in TRPC3 KO groups (*n* = 4) as shown in Figure 6B. The results further confirmed that TRPC3 was involved in OAG produced Ca^2+^ entry through ROCE upon GPCR activation. 2 μM Pyr10 significantly reduced the resting Ca^2+^ elevation produced by OAG treatment in WT but not in TRPC3 KO mice (Figure 6C). Unexpectedly, PyR10 produced no significant changes in Ca^2+^ transient amplitude and firing rate in both groups after exposure to OAG. The results indicated that Ca^2+^ entry through ROCE appeared to mainly influence resting Ca^2+^ in pacemaker cells and the lack of effect of PYR10 after OAG stimulation on the Ca^2+^ transient and pacemaker firing rate may reflect additional effect(s) of PyR10 beyond TRPC3. In support of the latter idea, 20 μM Pyr10 reduced the amplitude of the Ca^2+^ transient in TRPC3 KO mice without significantly changing pacemaker firing rate or resting Ca^2+^ level (data not show).

## Discussion

### TRPC3 and sino-atrial arrhythmias

In this study, we found that pacing-induced atrial fibrillation in angiotensin II treated mice was significantly reduced in mice lacking the TRPC3 gene (TRPC3^−/−^ mice). This suggests that TRPC3 channels may be involved in Ang II induced changes in the electrical properties of sinoatrial tissue. Recent evidence has implicated Ca^2+^ entry through TRPC3 as a pro-arrhythmic pathway (Harada et al., 2012). TRPC3 is known to be up-regulated in AF patients and experimental goat and canine AF models and also mediates a non-selective cation current in atrial fibroblasts (Harada et al., 2012). TRPC3 has also been implicated in dysfunction of SAN (Yanni et al., 2011) and atrioventricular conduction block (Sabourin et al., 2012). Collectively, all of these studies point to a functional role for TRPC3 activation in cardiac tissue and linkage to arrhythmogenesis.

Some ryrazole derivatives have recently been suggested to be relatively selective blockers for TRPC channels (Schleifer et al., 2012). In WT mice, we showed that Pyr10, blocked the membrane depolarization of SAN cause by Ang II and attenuated the increase in pacemaker firing rate induced by Ang II (Figure 5). A higher concentration of Pyr10 (20 μM) induced slower and irregular pacemaker activity without changing the upstroke of the action potential (Figure 5D). The latter result suggests that Pyr10 did not block L-type Ca channels which provide the main cation current for the upstroke of pacemaker action potentials. (Irisawa et al., 1993) This effect of Pyr10 was similar to our previous observation using a different SOCE inhibitor (SKF-96365) (Ju et al., 2007). In contrast, Pyr10 did not cause any significant changes in slowing pacemaker action potentials recorded from sinoatrial tissue from TRPC3^−/−^ mice (Figure 5H). These results suggest that Pyr10 is mainly active on a SAN diastolic membrane current, and is in accord with the idea that TRPC3 activation can provide a non-selective cation current in pacemaker cells (Eder and Molkentin, 2011). Intracellular Ca^2+^ measurements also showed that GPCR activation by Ang II or the DAG analog, OAG, caused an increase in both resting Ca^2+^ and Ca^2+^ transients that were associated with increased pacemaker firing rate in WT mice (Figure 6). The increased resting Ca^2+^ seemed directly related to ROCE as resting Ca^2+^ was significantly reduced in the presence of ROCE blocker, Pyr10 (Figure 6C). In contrast, such changes were absent in the TRPC3 ^−/−^ group. These results provide further evidence that TRPC3 is involved in regulation of intracellular Ca^2+^ and ROCE mechanisms in SAN (Figure 6).

### Interaction between IP_3_R and SOCE in cardiac pacemaker tissue

It has become generally accepted that Ca^2+^ release from the SR contributes to pacemaker activity through its influence on NCX currents (Ju and Allen, 2001; Vinogradova et al., 2005; Maltsev and Lakatta, 2008). Alterations in Ca^2+^ metabolism and consequent changes in Ca^2+^ dependent currents (such as NCX) have also been implicated in failure of pacemaker function and cardiac arrhythmias (Du and Nathan, 2007; Maltsev and Lakatta, 2007).

We previously reported that IP_3_Rs are expressed in murine SAN and that the predominant isoform is the Type II IP_3_R (IP_3_R2) (Ju et al., 2011). Importantly, the modulation of pacemaker firing and intracellular Ca^2+^ by IP_3_R-agonists and antagonists was abolished in IP_3_R2 KO mice, demonstrating a clear functional, modulatory, role for IP_3_R2 in SAN. The present study also supports the idea that Ca^2+^ release via IP_3_Rs can modulate heart rate, depending on the activation of G-protein coupled receptors and the phospholipase C-IP_3_ signaling pathway.

In non-excitable cells and smooth muscle, it has also been suggested that SOCE are coupled to IP_3_Rs (Putney, 1986). Since the discovery of TRPC channels and their candidacy for the SOCE, interactions between IP_3_Rs and members of the TRPC family have been reported (Vazquez et al., 2004). For example, it has been shown that IP_3_R2 interacts with TRPC3 forming a protein complex that possibly underlies the enhanced SOCE activity seen in gravid uterine endothelium (Gifford et al., 2006). In the present study, we show that Ca^2+^ influx through SOCE in the SAN can be modulated by IP_3_ agonists(ET-1, IP_3_-BM) and IP_3_R2 antagonists (2-APB) through mainly their effect on increasing Ca^2+^ release and hence reducing store content (see Figure 4). Since some IP_3_Rs appear to be localized near the surface membrane, where TRPC3 is also located (Ju et al., 2007, 2011), it is possible that Ca^2+^ release via IP_3_Rs could interact with these ion channels in the surface membrane (and/or affect their trafficking). IP_3_R expression has been shown to increase in heart failure (Marks, 2000) and in atrial fibrillation (Yamada et al., 2002), which raises the possibility that this IP3-TRPC signaling system may become more important in pathological conditions (Worley et al., 2007; Ju et al., 2012).

### SOCE over ROCE in cardiac tissue

As described above, SOCE and ROCE may be linked phenomena but the extent to which these mechanisms cross-communicate/activate is unclear -especially after GPCR activation of PLC which triggers formation of IP_3_ and DAG (Liao et al., 2009). While it has been established that TRPC3 is involved in ROCE (Hofmann et al., 1999), it remains uncertain whether TRPC3 can also contribute to SOCE (for review see Cahalan, 2009) (Zagranichnaya et al., 2005; Dehaven et al., 2009).

We showed that TRPC3 expressing pacemaker cells also express the ER-Ca^2+^ sensor protein, STIM1. The latter may provide the missing molecular signaling link between Ca^2+^ release via IP_3_Rs, store depletion and the activation of SOCE(Lewis, 2007; Yuan et al., 2007). However, it remains unclear whether STIM1 directly regulates the TRPC channels related to SOCE (Worley et al., 2007; Yuan et al., 2007; Dehaven et al., 2009). In addition to TRPCs, Orai1 is a protein that may produce a specific SOCE pathway and appears as a Ca^2+^ release activated Ca^2+^ current (Icrac). While we found that Orai1 is expressed in mouse pacemaker cells (not shown), TRPC3 was more dominant at both mRNA and protein levels (Ju et al., 2007). It has been suggested that TRPC3 and Orai1 may form a complex that mediates both SOCE and ROCE and the conversion from ROCE to SOCE may be mediated through STIM1 binding (Liao et al., 2008).

### The limitation of current study

The current study does not rule out the possibility that Pyr10 has effects on currents other than TRPC3, such as I_*f*_. In addition, it would be desirable to examine if other molecular components, such as NCX, SERCA, etc. that regulate intracellular Ca^2+^ are changed in TRPC3^−/−^ mice. Despite these limitations, we shown that Ca^2+^ entry through both SOCE and ROCE occurs in murine SAN and appears to be regulated by the GPCRs in a GPCR≫ PLC ≫ IP_3_R/DAG ≫ Ca^2+^ signal transduction cascade. We suggest that TRPC3 channels might provide a potential target for future treatment of SAN dysfunction and AF as it links both receptor activation and intracellular Ca^2+^ signaling.

### Conflict of interest statement

The authors declare that the research was conducted in the absence of any commercial or financial relationships that could be construed as a potential conflict of interest.
